# How Can Health Systems Better Prepare for the Next Pandemic? Lessons Learned From the Management of COVID-19 in Quebec (Canada)

**DOI:** 10.3389/fpubh.2021.671833

**Published:** 2021-06-18

**Authors:** Hassane Alami, Pascale Lehoux, Richard Fleet, Jean-Paul Fortin, Joanne Liu, Randa Attieh, Stéphanie Bernadette Mafalda Cadeddu, Mamane Abdoulaye Samri, Mathilde Savoldelli, Mohamed Ali Ag Ahmed

**Affiliations:** ^1^Center for Public Health Research of the University of Montreal, Montreal, QC, Canada; ^2^Department of Health Management, Evaluation and Policy, University of Montreal, Montreal, QC, Canada; ^3^VITAM Research Centre on Sustainable Health, Laval University, Quebec, QC, Canada; ^4^Department of Family Medicine and Emergency Medicine, Faculty of Medicine, Laval University, Quebec, QC, Canada; ^5^Research Chair in Emergency Medicine Université Laval-CHAU Hôtel-Dieu de Lévis, Lévis, QC, Canada; ^6^Department of Social and Preventive Medicine, Faculty of Medicine, Laval University, Quebec, QC, Canada; ^7^Faculty of Medicine, University of Montreal, Montreal, QC, Canada; ^8^Research Centre of the University of Montreal Hospital Centre, University of Montreal, Montreal, QC, Canada; ^9^Faculty of Law, University of Montreal, Montreal, QC, Canada; ^10^School for Advanced Studies in Public Health (EHESP), Rennes, France; ^11^Research Chair on Chronic Diseases in Primary Care, Sherbrooke University, Chicoutimi, QC, Canada; ^12^The Institute of Tropical Medicine, Antwerp, Belgium

**Keywords:** health system resilience, Quebec (Canada), health crisis management, health system readiness, health system preparedness, public health policy, governance, pandemic (COVID-19)

## Abstract

The magnitude of the COVID-19 pandemic challenged societies around our globalized world. To contain the spread of the virus, unprecedented and drastic measures and policies were put in place by governments to manage an exceptional health care situation while maintaining other essential services. The responses of many governments showed a lack of preparedness to face this systemic and global health crisis. Drawing on field observations and available data on the first wave of the pandemic (mid-March to mid-May 2020) in Quebec (Canada), this article reviewed and discussed the successes and failures that characterized the management of COVID-19 in this province. Using the framework of Palagyi et al. on system preparedness toward emerging infectious diseases, we described and analyzed in a chronologically and narratively way: (1) how surveillance was structured; (2) how workforce issues were managed; (3) what infrastructures and medical supplies were made available; (4) what communication mechanisms were put in place; (5) what form of governance emerged; and (6) whether trust was established and maintained throughout the crisis. Our findings and observations stress that resilience and ability to adequately respond to a systemic and global crisis depend upon preexisting system-level characteristics and capacities at both the provincial and federal governance levels. By providing recommendations for policy and practice from a learning health system perspective, this paper contributes to the groundwork required for interdisciplinary research and genuine policy discussions to help health systems better prepare for future pandemics.

## Introduction

“*Now, when the war is abolishing landmarks of every kind, is the opportunity for using experience in a clear field. A revolutionary moment in the world's history is a time for revolutions, not for patching” (William Beveridge, 1942)* (1).

The world has been taken aback by the magnitude of the COVID-19 pandemic (2, 3). High-income countries have faced an unprecedented situation since the 1918 “Spanish” influenza pandemic (4). In the absence of effective treatments and vaccines, governments sought to deploy emergency measures to contain the spread of the virus. These included closing borders, banning social gatherings, shutting down economic activities, closing schools, etc. (5–11). The exceptional nature of the situation challenged all countries in their efforts to decide, implement, and operationalize containment-related measures (5, 11–13).

Despite the resources and infrastructures at the disposal of high-income countries, the spread of SARS-CoV-2 has raised questions about the ability of health and social services systems (HSSS) to cope with a health crisis of such magnitude. According to the Director-General of the World Health Organization (WHO): “*Even countries with advanced health systems and powerful economies have been overwhelmed”* by the COVID-19 pandemic (14). Although the crisis is still ongoing, it is essential to start research on what could have affected the system's preparedness for this pandemic (15–17). The current crisis offers a unique opportunity to analyze how HSSS can better prepare for the increasingly systemic and globalized challenges to come in the future (18).

The objective of this article is to describe and discuss the experience of the HSSS in the Canadian province of Quebec in the management of COVID-19 crisis.

## Methodology

Quebec is the second largest Canadian province and the one that suffered the most from the first wave of COVID-19 in terms of incidence and mortality (Table 1). It is therefore a “textbook case” of interest for addressing the preparation of HSSS for the management of health crises.

**Table 1:** COVID-19 data for selected provinces (Quebec, Ontario, British Columbia) and at the Canadian level, as of May 20, 2020.

	**Confirmed cases**	**Active cases**	**Deaths**	**Deaths in residential and long-term care centers**	**Hospitalizations**	**Tests**
**Quebec** (19–21)	45,773 (Other sources: 44,775)	10,641	4,520 (Other sources: 3,718)	3,162	1,500 (175 in intensive care)	317,902
**Ontario** (22, 23)	23,774	3,622	1,962	1,452	991 (160 in intensive care)	454,094
**British Columbia** (20–26)	2,467	317	149	–	43 (10 in intensive care)	107,152
**Canada** (20–26)	80,123	33,379	6,030	–	–	–

*Data is from different sources as an integrated Canadian database is unavailable. Some of the data differs between sources. This may be explained by the methods used or time lags for collection and compilation, which makes comparison difficult. Values should be considered with caution. That said, the different sources show the same overall trends*.

To document the process, different sources of data were used. We searched Google and Google scholar, as well as the websites of the provincial and federal governments, and the organizations directly or indirectly involved in the management of the crisis in Quebec and Canada (more details on these organizations are provided below) (27). Given the exceptional context that did not allow for in-depth empirical data collection, we also collected testimonials from the field, including first-hand clinical and managerial experiences, as well as leveraged our experiences to bring about reflections. These testimonies and reflections are relevant to this paper's aims because the authors include researchers specialized in various aspects of the Quebec HSSS (e.g., health crisis, public health, health services and policy, health technology assessment) as well as practitioners (e.g., public health physicians, emergency physicians), some of whom were on the front lines during the pandemic. The analysis covered the first wave containment period, which occurred between mid-March and mid-May 2020.

The whole corpus of data was compiled and coded with Endnote. In order to structure our analyses, we relied on the conceptual framework of Palagyi et al. (28) on HSSS preparedness for emerging infectious diseases (EID), which was developed following a review of the literature on the determinants of HSSS preparedness for EID. This framework covers four “hardware” and two “software” constructs. The four “hardware” constructs include: (1) surveillance, (2) workforce, (3) infrastructures and medical supplies, and (4) communication mechanisms. The two “software” constructs focused on human and institutional relationships, norms and values, and consist of: (1) governance, and (2) trust (28). In this context, “hardware” refers to the tangible organizational, financial and legal dispositions that structure, and frame the entire HSSS, as well as the resources related to the clinical and service delivery requirements. “Software” refers to the ideas, values, interests and norms, as well as the power dynamics and affinities that underlie the relationships between the actors, elements and levels of the system (28, 29). The “software” components enable the whole “hardware” to be maintained. We chose this framework for two reasons. First, it highlights the interdependence and interconnections between the different constructs. Second, its originality lies in the fact that it emphasizes the central role of governance and trust in achieving and sustaining HSSS preparedness for EID (28). Despite its limitations, our approach to the crisis management process allowed our team to bring conceptual and empirical clarity to the key issues raised by each component of the analytical framework (27, 30, 31).

Before presenting our findings, a brief overview of the broader Canadian context wherein the Quebec HSSS evolves has been presented in the following section. This information is particularly useful for readers unfamiliar with the wide variety of roles of federal and provincial governments in healthcare policies and to better understand the facts and observations reported on crisis management.

## Overview of the Quebec Health and Social Services System

Canada is a federal state constituted of 10 provinces and three territories that have significant powers over their own policies (32, 33). While health policies are under provincial jurisdiction (11, 32), the federal government contributes to the funding of the provincial health sector on the condition that the provinces comply with the Canada Health Act (34). The Public Health Agency of Canada (PHAC) and the Department of Public Safety and Emergency Preparedness (PSEPC) both operate under the Federal government (11). Canada generally follows the recommendations of the WHO, including the “International Health Regulations” (11, 35, 36).

The Quebec HSSS possesses several features of the Beveridge model, like Canada and the United Kingdom (37, 38). Its governance lies at two main levels: (1) the Ministry of Health and Social Services (MSSS) is responsible for defining, regulating, and coordinating key strategic directions, priorities and objectives and allocates resources to these ends; and (2) 22 Integrated Health and Social Services Centres (CISSS or CIUSSS for those including an academic mission) provide most primary care and services and some specialized services, four university teaching hospitals provide specialized and subspecialized care and services, and two university institutes provide specialized cardiology services. For their part, residential and long-term care centres (CHSLD), mainly under the tutelage of CISSS/CIUSSS, are responsible for providing accommodation and medical and social care services to adults with limited functional and/or psychosocial autonomy (39).

This governance structure is the result of a significant centralization reform implemented in 2015. It consisted in major mergers, on a territorial basis, of health and social services organizations, from 182 to 34, resulting in large complex organizations, the CISSS/CIUSSS, with 12,000–15,000 employees each (40). Following the logic of austerity, a significant decrease in the number of health managers and executives by about 20% (~1,300 positions abolished at different levels) followed (40, 41). While public health high-level capacities were maintained at the MSSS, most public health activities that were historically more independent were integrated within the CISSS/CIUSSS structures (42). Because the public health budget was cut by 33%, several prevention, promotion, protection and surveillance functions were limited, and the public health mission related to the organization of health services was eliminated (42).

Within the Quebec HSSS, physicians are paid by the public health insurance system, provided they do not have private sector activities. A very small percentage (about 2%) of physicians, called non-participants, have a private practice paid directly by patients and/or private insurance (43). Other health care providers (e.g., nurses) are mainly salaried employees of their own organizations. Professional colleges and federations play an important role in the supervision of practice (e.g., Quebec college of physicians -CMQ-, Order of nurses of Quebec -OIIQ-) and the defense of their members' rights (e.g., Federation of medical specialists of Québec -FMSQ-, Federation of general practitioners of Quebec -FMOQ-).

Two public knowledge-based agencies play a key role: (1) the Quebec National Public Health Institute (INSPQ) is tasked with, among other things, monitoring the health and well-being of the population and its determinants, assessing the impact of public policies on population health, providing screening, laboratory and quality maintenance services, and supporting the MSSS and the regional public health organizations in carrying out their responsibilities (44); and (2) the National Institute for Excellence in Health and Social Services (INESSS) is tasked with, among other things, assessing the clinical benefits and costs of health and social service innovations (e.g., drugs, technologies, therapies), developing clinical recommendations, and guidelines in accordance with best practices and responding to requests for advice and recommendations from the MSSS (45). As we will see below, these two organizations were extremely active throughout the pandemic.

## Findings

Our analysis adopted a theoretically-informed narrative structure that integrates a factual chronology of key events related to the COVID-19 pandemic until the end of lockdown, after the first wave, to better contextualize the lessons learned (Table 2) (47). It follows the structure of Palagyi et al.'s (28) framework composed of “hardware” constructs that are: (1) surveillance; (2) workforce; (3) infrastructures and medical supplies; and (4) communication mechanisms, followed by “software” constructs including: (1) governance; and (2) trust (28). The findings shed light on lessons learned and ways forward to inform decision-makers and researchers who wish to develop more in-depth analyses in different provinces or countries.

**Table 2:** A brief chronology of key events related to the first wave of COVID-19 in Quebec (46).

**Date**	**Key events**
February 27	Announcement of the first case of COVID-19 in Quebec
March 11	WHO declares pandemic status for COVID-19 Isolation of travelers, prohibition of meetings of more than 250 people
March 14	Declaration of the state of emergency in Quebec for the first time in its history
March 15	Physical distance measurements (1 m) Closure of several public places (e.g., bars, theaters, swimming pools) Citizens are called upon to limit their movements
March 16	Canada declares partial border closures Closure of daycares and schools in Quebec
March 17	Quebec's National Assembly suspends its work
March 18	First death reported in Quebec Increased capacity for screening, including travelers and their symptomatic contacts
March 19	Opening of the first “mobile clinics” for COVID-19 screening
March 21	Prohibition of all indoor and outdoor meetings
March 22	Closing of commercial centers, hairdressing salons and other businesses
March 23	Mandatory containment in CHSLDs and private residences for seniors New screening priorities include symptomatic patients, health care providers, and CHSLD residents
March 24	Shutdown of all services, except those considered essential
March 27	Restriction of travel between Quebec regions The physical distancing requirement is increased from 1 to 2 m The Montreal metropolis declares itself in a state of health emergency
April 4	The state of community transmission of the virus is declared
April 21	The Quebec Prime Minister requests the intervention of the Canadian army in the CHSLDs
May 21	The objective of 14,000 people screened per day is reached
May 22	Gradual decontainment. Authorization of outdoor meetings for up to 10 people from up to 3 households

## Hardware

### Surveillance

The pandemic highlighted several issues related to surveillance and monitoring for crisis management purposes.

#### Missing and/or Inconsistent International Data

In accordance with the Public Health Act, Quebec declared a state of public health emergency on March 14, 2020 due to COVID-19. At this stage, the assessment of the seriousness of the situation and the ability of the Quebec HSSS to respond in time depended in part on the quality and reliability of the information provided by the countries affected first and by the WHO. The fragmented and sometimes contradictory information made available in other countries was a major challenge to manage at the onset of the crisis (48). Countries reported statistics in different ways, making comparisons difficult (48). For example, several countries did not account for deaths that occurred outside the hospital or that had not been confirmed by laboratory results (49). In late March, Quebec also changed its strategy by counting probable cases as confirmed cases.

#### Lack of Reliable Monitoring and Information System

When the first case of COVID-19 was reported in Quebec, the Province lacked a reliable health monitoring and information system. There were significant difficulties in bringing first line data on the evolution of the situation to the MSSS in real time: cumulative number of cases and new cases, number of hospitalizations, number of people cured, number of people who died, number of people screened, détails on their geographic location, age, gender, and clinical history, etc. The MSSS was unable to obtain the data in the field. Discrepancies between data communicated by the government and the real situation on the ground were observed (e.g., 33 deaths occurred at the University Institute of Geriatrics of Montreal while the official count of the MSSS was 5 deaths) (50). In addition, the COVID-19 cases were recorded primarily by fax (51).

The lack of information may also have delayed and/or complicated the ability to plan and organize actions, and to adapt strategies according to the needs and capacities of health organizations to meet demand: redeployment of clinical staff; preparation of hospital beds; procurement and distribution of mechanical ventilators, protective equipment (e.g., masks, gloves, gowns) and testing materials (e.g., swabs, reagents); management of quarantines; and the development of clinical guidance for intensive care and resuscitation decisions (52). By the end of May 2020, more than half of the COVID-19 deaths had occurred in residences for elderly people (Table 1) (19, 53, 54). There were many surveillance gaps in these centers (e.g., number of deaths per residence, available places, number of tests conducted, number of staff missing), despite the fact that they are high-risk sites for infection and spread of the virus. For several weeks, the government was unable to obtain reliable information on the situation in these centers. Yet, in its 2012 report, the Auditor General (AG), an independent evaluation institution, had pointed out the lack of information on the available resources and the situation in CHSLDs (e.g., quality of services, evaluations and audits carried out, resource management, portrait of residents' needs). To address this weakness, the AG had recommended the implementation of a harmonized evaluation system and a reliable information system (55). Nevertheless, these recommendations were not implemented. In this regard, harmonizing information in the Quebec system of caring for people with loss of autonomy is a very complex exercise. Indeed, information is collected by different actors (e.g., public and private CHSLDs, intermediate resources, residences for the elderly) through information systems that are not completely integrated (52, 56). According to Denis et al. (57), “*The crisis has highlighted the unacceptable lag of Québec regarding the collection, curation, and analysis of data on population health and its determinants*” (57).

Screening was identified as an essential strategy to obtain an accurate portrait of the number of cases, their evolution and their distribution across the territory. However, the widespread implementation of automatic screening proved to be impossible, in part because of the lack of testing equipment (e.g., swabs, reagents) due to global competition and because of the sub-optimal laboratory capacities and functioning. By mid-March 2020, the capacity of 6,000–7,000 tests per day was reached for a population of ~8.5 million people. The number of reported cases was likely to be underestimated. Several deaths caused by the virus could not be counted, either because they were misdiagnosed (e.g., pneumonia) or because they occurred in the community. Deceased persons were not automatically tested for COVID-19 and suspicious cases were not necessarily included in official statistics. The challenge then was to identify those who actually died from COVID-19 and those who died due to a lack of care required for other needs and pathologies (58).

#### Difficulty in Accessing Data for Research and Evaluation

The crisis also highlighted the problem of access to health data for research and evaluation purposes that has persisted for years in Quebec. Researchers deplored the quasi-impossibility of accessing quality data to develop models and solutions different from those promoted by the government, and also to cross-check the latter (57). The procedures for obtaining authorization to access data are complex (e.g., delays, people must be physically present on site to have access to the data).

### Workforce

The pandemic highlighted and/or exacerbated a number of challenges related to the availability, distribution, training, safety, and protection of the health care workforce.

#### Insufficient Workforce in Public Health

The public health sector experienced a significant shortage of human resources to perform surveillance activities, epidemiological investigations (e.g., identifying places frequented and people in contact with COVID-19), as well as promotion and prevention activities (e.g., containment, social distancing, hygiene practices). The need was partially offset by rapidly trained volunteers. Indeed, because the public health sector had suffered a 33% reduction in its budget in 2015, many experts were no longer available (e.g., workforce reductions, resignations, retirements). The proportion of public health funding in Quebec is 2.2%, which is less than the recommended 5–6% (42). Likewise, the Canadian public health capacities and infrastructures to respond to health crises had weakened over the years, despite recommendations following the 2003 Severe acute respiratory syndrome (SARS) outbreak (e.g., Naylor report) (59, 60).

#### Insufficient Workforce in Hospitals and Clinics

The availability and distribution of health care providers was a major issue, particularly given the vastness of Quebec's territory (1,667,712 km^2^). Rural and remote regions experienced problems with recruiting and retaining health professionals for many years. At the time of the crisis, rural hospitals were not fully prepared to meet the demand if the situation had deteriorated further within their territories (61, 62). In addition to aeromedical evacuations between regions, telehealth was rapidly deployed to ensure coverage of services and sharing of expertise (61, 63, 64). Clinicians in preventive isolation or quarantine were able to continue providing services remotely (e.g., teleconsultation, prescription of medicines). COVID-19 has served as an accelerator for the widespread use of telehealth. However, in a context where Quebec did not have a functional and integrated telehealth network throughout its territory, this rapid deployment may have raised and/or exacerbated a number of challenges in terms of human resources (e.g., planning and organization of teleconsultations and telemonitoring of patients) (37, 65, 66).

In addition, to make up for this deficit, in view of the exceptional situation as defined in the Quebec Public Health Act, the government authorized private sector physicians to practice in the public sector while continuing their private practice (67). Private clinics and physicians were also asked to take over certain procedures and surgeries that had accumulated delays due to COVID-19, thereby authorizing a “hybrid” (public-private) medical practice, until now prohibited for Quebec physicians.

#### Insufficient Workforce in Long-Term Care Facilities

The high mortality caused by COVID-19 in CHSLDs signaled acute problems of availability and distribution of qualified human resources and organization of services (52, 68, 69), an issue already raised in the 2012 AG report (55). In 2018–2019, human resources available in the CHSLDs were able to meet only 71% of the needs for routine care and services (70). A 2015 study also found that long-term care needs were significantly higher than the available resources (71). For example, due to the lack of nursing staff in CHSLDs who could provide palliative care, patients were forced to be transferred to already overstretched hospitals. To overcome this gap, more than 2,000 medical specialists and 400 family physicians were mobilized in CHSLDs to care for and/or assist the staff on site (e.g., clinical care, hygiene, nutrition and hydration) (70). However, because hospital staff were at risk of spreading the virus upon their return to the hospital, their redeployment to CHSLDs was not unanimously accepted. Personnel from the Canadian Armed Forces and the Canadian Red Cross were also called in as reinforcement in CHSLDs (70, 72). In the same vein, the government has set up a web portal entitled “Je contribue !” (I Contribute!) to facilitate the recruitment of people who wished to offer their services to the HSSS, especially in CHSLDs. The platform had several deficiencies such as file tracking and volunteer deployment (70). The government had also to review the selection criteria to expand the available labor pool (e.g., clinicians with foreign diplomas, students).

#### Ensuring Workforce Training, Safety and Protection

With COVID-19, the challenge was to provide accelerated training on clinical practice and management of patients with infectious diseases in health organizations in Quebec. Protocols and training (e.g., webinars, accelerated online courses) were quickly developed at the local level. However, this multiplication of protocols and training may have been a source of concern due to quality variations in practices, including risks to the quality of care, as well as to the protection and safety of patients and health care providers. According to the Canadian Medical Association, 19% of those infected with COVID-19 were health care workers, even though they represent only 8% of the country's workforce (73).

Under the state of emergency, managers were given the authority to suspend leaves of absence and vacations for health care staff, redeploy/reassign them to other settings (e.g., CHSLDs) or impose overtime. Some workers were assigned to tasks for which they had not been adequately prepared or trained (52). This overburdening, caused by difficult working conditions, was associated with increased burnout, anxiety, alcohol consumption and mental health problems among health care providers (74–76). According to Statistics Canada, the average overtime worked by nurses in Quebec increased from 6.2 h in May 2019 to 16.9 h in May 2020 (77). In early April 2020, more than 10,000 health care workers were absent (e.g., quarantine, exhaustion, resignation), including more than 4,000 infected with the virus (78). This substantial outcome strained the capacity for action in the field.

#### Misaligned Incentives and Professional Jurisdictions Issues

The government offered financial incentives to encourage health care providers to work in certain settings (e.g., CHSLDs) and/or to provide certain services (e.g., teleconsultation). From the beginning, the reimbursement of physicians for telehealth procedures had been authorized, including telephone consultations. A bonus of 8% was granted to people working in direct contact with infected patients in emergency rooms, intensive care units, clinics, CHSLDs and ambulances. For their part, laboratory technicians and cleaning staff were entitled to a 4% bonus. Beneficiary attendants, particularly in CHSLDs, received a CA$4/hour increase on an average hourly rate of approximately CA$20/hour. However, these bonuses raised much criticism. It was stressed that to maintain collective commitment on the ground, all professionals involved in the crisis should benefit from such bonuses (79).

Jurisdictional and mission frameworks (e.g., reserved activities, authorization to practice in certain areas) have been adapted to allow greater agility in responding to needs and rapid redeployment of staff to different settings (e.g., hospitals, CHSLDs), in particular by decompartmentalizing areas of practice and transferring tasks between professional groups (72). However, various professional groups have not been able to apply the full extent of their knowledge and skills (e.g., dentists, dietitians, speech therapists) (72). This is not surprising given the preexisting sealed scopes of practice, where institutional arrangements have isolated many clinical practices (72).

### Infrastructures and Medical Supplies

The global scale of the pandemic has led to fierce competition between countries for the acquisition of medical equipment and drugs (e.g., some countries took hold of shipments, embargoes, defective equipment). In addition, the lack of a global dashboard on material resources and production capacities was one of the first challenges for decision-makers. This situation may have had a significant impact on other levels of care and services, not necessarily related to COVID-19.

#### Medical Equipment Shortage

The urgent need for medical equipment (e.g., masks, gloves, mechanical ventilators, screening kits) showed the strong dependence of Quebec, and Canada, on external suppliers (e.g., China, USA). Strategic stocks quickly proved to be insufficient to confront the crisis (11). In 2007, in a report produced after the Avian influenza (H5N1) and the SARS pandemics, INSPQ had already warned of the shortage of masks (80). The Institute had pointed out that Quebec was heavily dependent on masks produced in the USA, which could cause supply hurdles in the event of a health emergency. During the COVID-19 crisis, the shortage occurred earlier than expected and forced staff in some organizations to wash and reuse disposable gowns. Some organizations had to extend the use of N95 masks or even recycle them (81). In this context, Quebec was the victim of an attempt to swindle CA$45 million for the purchase of 5 million N95 masks that did not exist (82).

#### Drugs Shortage

Concerns were raised about the lack of essential drugs, including opioids and anesthetics used in the care of COVID-19 patients (83). *Propofol, morphine, curare*, and other sedatives and anesthetics were at risk of shortages and/or stock-outs. Other essential drugs for common conditions also experienced shortages, particularly due to over-prescribing or stockpiling of exaggerated quantities by patients and hospitals at the beginning of the COVID-19 crisis (e.g., purchases for several months of inventory instead of the usual few days/weeks) (84). For example, *Salbutamol*, used for respiratory problems (e.g., asthma), was officially classified as being in shortage in Canada as of April 17, 2020 (84). As for masks, the question of the “sanitary independence” of Quebec and Canada arose. The country depended upon external market for drug supply (e.g., China, India). COVID-19 showed the limited room for maneuver and the lack of available levers to address local drug needs in a global health crisis context (85, 86). In the past, Canada had already experienced drug shortages (e.g., Sandoz) (87, 88) and experts had pointed out that these shortages were “*more than just background noise*” (87).

#### “Collateral” Consequences and Ethical Dilemmas

While the HSSS's attention and actions were concentrated on the crisis, the care and follow-up of patients suffering from “common” health problems became critical (89, 90). By mid-April 2020, the decrease in consultations for acute heart attacks was 40% to 60% (91, 92). Visits for certain vascular events (e.g., minor strokes, transient ischemic attacks) decreased by as much as 80%. Deaths and serious sequelae ensued from recurring postponement of non-urgent hospital activities and increasing public fear of contracting the virus when visiting a health care facility (93). For example, given the patients' reluctance to consult and/or the difficulty of accessing services on time, the number of complications related to cardiac infarction registered in 1 month was equivalent to that of the entire previous year (94, 95). Similar findings were observed in oncology (93, 96). Between March and June 2020, oncology surgeries decreased by 16% (around 1,500 surgeries), and more than 4,000 people with cancer have not been diagnosed (97). For the President of the Quebec Association of Hematologists-Oncologists: “*The reality is that cancer kills more than COVID-19 in Quebec*” (own translation) (98). In mid-May 2020, the number of surgeries postponed since the beginning of the crisis was ~68,000 according to the MSSS (81,000 according to the FMSQ) (99). Possible complications related to the lack of mental health, addiction and psychosocial services were also raised (100–102).

The lack of medical equipment and drugs raised ethical dilemmas, especially in the absence of clinical guidelines and ethical frameworks to inform decisions (e.g., resource allocation, restrictive measures). At the outset of the crisis, many clinicians had called for clear protocols, particularly in determining the type of patients to be prioritized if medical equipment was lacking (e.g., mechanical ventilators, beds). Health care providers expressed the need for support in situations where the moral burden could prove untenable and to avoid arbitrary decisions. Shall they adopt a first-come, first-served principle? Should a younger patient be prioritized over an older one? Such feelings of facing a “death lottery” were reported in other countries as well (103).

## Software

### Communication Mechanisms

Communication in a crisis is multidimensional and was made even more complex by the prominence of social networks and digital media as a source of information.

#### Consistency and Transparency in High-Level Communication Strategy

Quebec authorities chose a centralized communication strategy, which featured a trio: the Quebec Prime Minister, the Minister of Health and Social Services and the National Public Health Director (a public health physician). The trio held daily press briefings to inform the population about the evolution of the pandemic, the government orientations and their implications. The National Public Health Director's communications were generally well-received (104). The informal tone he adopted contributed to the wide dissemination of public health instructions on social networks and in the media. The trio-led communication strategy proved to be relatively effective (105). In addition, the media gave clinical and academic experts important space to analyze the situation and answer questions from the public (105). The scientific “authority” figures helped to depoliticize the management of this crisis first wave. Elected officials' decision to suspend partisan disputes and support the government may also have increased the effectiveness of the communication strategies deployed (106).

However, some communication failures have been observed. For example, contradictions as to the effectiveness or otherwise of masks to contain the spread of the virus in the general population have generated doubts and confusion about the appropriateness of this measure (107, 108). Inconsistencies may have given more resonance to the message of anti-mask demonstrations that took place in Quebec (107).

Communication was also at times difficult between the government and health care providers. Many clinicians felt that the authorities were far from telling what was really happening on the ground, particularly with regard to HSSS capacity and the lack of beds, medical equipment and drugs. Others have reported that it was unacceptable to have the media as a primary source of information on COVID-19 (109). More transparency was called for and the large influx of information and directives from health organizations and the MSSS (e.g., clinical protocols, safety and biosafety, isolation, prioritization), that were sometimes contradictory, was pointed out. A lack of coordination and coherence in communication between the MSSS and the organizations thus complicated the work of clinicians and managers in certain situations.

#### Managing Communication in the Social Networks and Digital Media Era

The COVID-19 pandemic has occurred in a context where social networks and digital media are key sources of information for the population and journalists. In principle, the population could have had greater access to reliable information and messages from the government could also have been better understood (110). Yet, the digital era presented an unprecedented challenge for decision-makers. Indeed, communication has been made difficult by the proliferation of sensationalist reporting, unverified information (e.g., toilet paper, compulsive purchases), fake news and conspiracy theories (111). For example, telephone towers have been set on fire in Quebec by people who believe the 5G network was responsible for the spread of the virus (112). Misinformation has also led to xenophobic reactions against individuals from Asian or Jewish origins.

To mitigate communication problems, approximately half a million dollars (CA) was spent to raise awareness of COVID-19, specifically among the 16–35 year-old group (e.g., advertising, recruitment of influencers) (113).

### Governance

The pandemic highlighted several issues related to governance, both at the federal and provincial levels. It also raised the problem of bureaucratic complexity that characterizes the Quebec HSSS.

#### Federal-Provincial Governance Relationships

The Canadian federal government has put in place numerous emergency measures to support people who have lost their jobs or suffered from a drop in income, as well as students and businesses (11, 114). The provinces have implemented their own policies along similar lines. However, some historical tensions related to the division of powers between the federal and provincial levels have resurfaced. The federal government has invoked the possibility of mobilizing “The Emergencies Act,” which comes with extraordinary powers (11). Several provinces, including Quebec, opposed this avenue as it would have undermined their own efforts and actions (115). A similar scenario emerged when the federal government wanted to bring the CHSLDs under “The Canada Health Act” because of the high mortality rate (Table 1). The Quebec government opposed it because health care is under provincial jurisdiction. What happened in the CHSLDs has shown the extent to which elderly care policies have fallen into the “cracks” of a complex system of distributed responsibilities between the federal and provincial governments (11, 52). This period was characterized by incomplete information and uncertainty as to which level of government, federal or provincial, is responsible for which policy (116). These unresolved questions posed challenges for the coordination of certain actions at the national level. In this context, PHAC has decided to publish guidelines and recommendations that the provinces may decide to adopt or not (116). As Willi et al. stress, traditional modes of governance in a federal state can be threatened, or at least weakened, in times of crisis, particularly with regard to different and conflicting actions and priorities at the federal and provincial levels (47). Given Canada's political history, seeing such tensions resurface is not entirely surprising.

#### Quebec Governance

Governance challenges in Quebec have also been observed. For example, clarifying roles and aligning positions between the provincial government and large municipalities was an important issue. The Montreal region, where about half of the cases were located by the end of April 2020 (117), took its own actions (e.g., setting up mobile test booths). Such “unilateral” actions were perceived as creating problems of synergy and coordination, and called into question issues of equity throughout the province (e.g., access to screening for the entire population of Quebec). In early June 2020, the government set up an “Intersectoral Recovery Table of the Montreal Health and Social Services Network” whose role was to increase synergy and coordination between stakeholders involved in crisis management in the metropolis.

At the beginning of the pandemic, INESSS and INSPQ faced coordination challenges in terms of the subject areas they would, respectively, cover. Different scientific recommendations could have been published on similar or related subjects, which is problematic in a crisis context. In addition, divergent positions between INESSS, INSPQ and the government could have increased confusion. INESSS concluded that safety, quality and efficacy criteria (e.g., scientific validity, clinical, and societal relevance) were not met to support the use of certain technologies (118). These rapid responses were issued in a context where the government was under pressure from the media and other actors, and needed to manage public expectations. In the same vein, INSPQ and the MSSS held different positions on whether wearing masks should be made mandatory in public places (107, 119). In addition, the government directed the school system to maintain original class sizes, contrary to the INSPQ's recommendation to reduce the number of students per class in order to reduce contacts (120). The government also did not follow the recommendations of public health experts to close daycare centers or to retroactively isolate travelers, especially health care providers, returning from abroad (121). The lack of transparency in the data used as the basis for some government decisions was noted. The government refused to make public advice and/or recommendations received from its experts (e.g., the National Public Health Director), which would have set more clearly apart which decisions were political and which derived from public health evidence (57, 121, 122). Such evidence-based decision-making concerns were also reported in Britain where many experts called on the British government to make the scientific evidence, data and models used for COVID-19-related decisions public and transparent within 72 h and on a regular basis (123).

#### Bureaucratic Complexity

Bureaucratic complexity and burden have often been underlined during the crisis, including by the Prime Minister himself. The centralization reform of 2015 created very large regional organizations that lacked the flexibility to ensure rapid and adapted responses to COVID-19 (72). This bureaucratic complexity within centralized governance revealed that the public health sector was locked into administrative structures that were not well-adapted to act quickly and effectively (42, 57). For example, authorities struggled to determine where the greatest workforce needs were, and health care providers were deployed to sites where there was no need for additional staff. Conversely, directives and guidelines sent by the MSSS to some CHSLDs have not “found” a local high-level manager capable of implementing them (53). Overall, despite the needs, thousands of clinicians and volunteers (e.g., internationally trained clinicians, community organizations) have not been fully mobilized and many have even been left behind (72).

### Trust

Trust intersects with all the dimensions discussed above and is crucial in times of crisis (124). Trust is multilevel, it involves several actors and dimensions, and it evolves in different ways across space and time.

#### Public Trust

Public trust in the decisions and strategies deployed to fight COVID-19 was present, yet partial. As mentioned earlier, the Prime Minister's popularity and the positive image of the National Public Health Director were reinforced during the first wave through the trio-based communication strategy. Social networks were a powerful channel for creating and establishing trust, but also for degrading it over time since trust remains fragile in times of crisis.

#### Uneven Trust Levels Between Health Care Providers, Organizations, and Government

Within organizations, contradictory information and the lack of equipment, protocols and clear guidelines may have undermined clinicians' trust in their hierarchy. For example, because of stolen equipment in some hospitals, cabinets containing masks were kept under lock by managers. This in turn reflected a lack of trust in clinicians who were nonetheless putting themselves at risk for the common good. The MSSS's decision to suspend the provisions of certain collective agreements was contested by health care providers. Managers were indeed granted full authority to assign or redeploy staff regardless of these contract agreements (e.g., nature of the position, task requirements, mobility, shift, mandatory overtime). They were also given the authority to suspend, cancel or refuse leaves of absence. Professionals were not been able to act as whistleblowers and denounce problems or dysfunctions in their organizations due to the fear of hierarchical reprisals (125). Such an authoritarian drift threatened an already fragile trust on the frontline. The government recognized the problem and set up the “*On vous écoute*” line (“We are listening to you”), enabling anonymous reports to the MSSS (126). In less than a week, more than 1,790 calls were received (127).

#### Decreased Level of Trust Toward Medical Unions

Before COVID-19, the 2015 reform proved controversial and weakened the confidence of a part of the population in the medical profession. Physicians' unions demanded and obtained significant salary increases, while social and community services and public health suffered important budget cuts. In a context of economic austerity, physicians' demands and gains were not well-received by the public, especially in light of high media scrutiny. In the midst of the crisis, when the Prime Minister asked medical specialists to help in CHSLDs and offered to pay them CA$211/hour —about five times the salary of a nurse for equivalent work—, the outrage of the public exploded. In the face of widespread protests, including from many physicians, the medical union and the government abandoned this idea.

#### Tracking Applications for COVID-19 and Mass Surveillance

Over time, smartphone applications (apps) were suggested as a potential solution to fight and monitor the spread of the virus (e.g., early detection, transmission control, and quarantine). In view of the various tracing apps deployed in other countries, this digital approach faced significant opposition, notably because of threats to privacy, risks of discrimination and other power abuses (128). Given the lack of trust, the government postponed its decision in order to benefit from more evidence, as well as legal and ethical guidance regarding these technologies. The aim was to provide clear and transparent answers to the public's questions (129). The feeling of abuse on the part of the population could undermine trust and transform it into mistrust (47).

## Recommendations and Avenues For Policy and Practice

In Quebec, the pandemic reemphasizes the “Overton window,” that is, the window of political possibilities and actions that become desirable, acceptable and/or unavoidable in light of a given context (130). The crisis has forced the social, economic and political landscape to evolve and transform in a surprising way, often against the tide of the “*new public management”* approach that dominated in recent years in Quebec (e.g., reduction in the public administration size, budget cuts in the public health sector) (131). It called into question the relevance and consequences of the recent reform toward a “minimal” state and a “managerialist” approach to public affairs (131). The “Overton window” implies that decision-makers should now adopt policies and actions in line with the needs, demands and realities of the times, but also in view of the challenges of the future (130). In this regard, the post-COVID-19 period represents an opportunity for bigger changes than what was previously acceptable and/or feasible in politics and for the public.

While it is very early to draw definitive lessons, some avenues for policy and practice can be identified to inform next steps for the Quebec government, as well as other jurisdictions and countries with comparable realities:

- **(Re)investing in and strengthening social policies**: the crisis has highlighted the importance of a socially-supportive state capable of designing and implementing the actions necessary to protect the population. Certain social policy choices focused mainly on austerity and disinvestment in public services (e.g., community services, social assistance, elderly) in recent years. These may have had a role in the varying impact (e.g., health, economic, social) of the virus on certain populations (e.g., disadvantaged neighborhoods, rural populations, the elderly in CHSLDs, minorities and indigenous populations).
- **Strengthening the health sector:** the health sector has been pushed to the critical limit of its capacity to respond to the population's needs for care and services. The offloading and/or postponement of other “less urgent” activities and procedures may have had a negative impact on people requiring other routine services (e.g., surgery, oncology, chronic diseases, primary care, mental health). It is partly the result of policies that have focused primarily on short-term operational and financial efficiency in public hospitals, at the expense of a true vision for investments that can strengthen the long-term resilience of this sector (132). Actions are required to strengthen an effective, accessible, integrated and overarching health system, especially by reinforcing the links between primary care and the public health sector (57).
- **Strengthening public health**: COVID-19, which includes national security issues, has reestablished public health - due to its cross-cutting strategic nature - as a key determinant of the resilience of the HSSS, the economy and society at large (133). It is necessary to restore the capacity of public health to perform its functions allowing it to prevent and manage crises of foreseeable magnitude of infectious diseases, but also for the current rise in chronic diseases or future natural and environmental disasters. To do so, there is a need to increase the portion of the health budget allocated to public health. This budget will need to be protected and significant enough to allow it to fulfill its mission (57). Public health should also have greater room for maneuver and autonomy, in contrast to the current situation where it is “*left within administrative structures that were ill suited to prioritizing action on major determinants of health*” (57).
- **Implementing a reliable health and social information system (HSIS)**: the absence of a reliable HSIS made it difficult to prepare the response and implement the necessary actions according to resources, needs and contexts. COVID-19 highlighted the vital importance of having mature and integrated information systems and infrastructures, quality data and rapid access mechanisms to inform policy and decision-making in health care delivery (75). This is an essential requirement for a “learning” HSSS (134). In addition, beyond technological infrastructures, organizational and bureaucratic culture should be aligned to cooperate effectively. The persistence of structural and sectoral silos could be a barrier to data access and sharing, and regulatory frameworks may crystallize such problems (e.g., the impossibility to access health data outside the physical walls of certain organizations).
- **Implementing an integrated, functional, and efficient telehealth network:** the various problems that have emerged with the rapid deployment and use of the technology can be explained, in part, by the fact that Quebec did not have established systems and procedures for telehealth. A solid Quebec digital health strategy is needed, especially to enable an integrated, functional and efficient telehealth network throughout the territory. This is also one of the conditions for building a functional and reliable HSIS.
- **Promoting interprofessional work, interdisciplinarity, and intersectorality in workforce training:** the crisis has highlighted the importance of integrating training modules on health crises into the curriculum (initial or continuous) of health care providers and managers. There is also a strong need to develop new training that values interprofessional, interdisciplinary, and intersectoral collaborations to avoid perpetuating silos (e.g., reserved acts, professional jurisdictions). To promote greater interdisciplinarity and intersectorality, the social sciences and humanities need to be integrated into the curriculum of health professionals (135). Training based on a “One Heath” approach - which emphasizes the interdependence of humans, animals and the environment - is becoming essential (136, 137).
- **Promoting the health and well-being of health care providers:** healthy health care providers are an essential condition for the provision of quality care and services to patients and the population (138). The crisis has shown that their health has often been taken for granted by decision-makers and managers, rather than as a priority requiring well-defined and coherent strategies and actions (139). It is necessary to ensure that the conditions are met to protect them, both in a context of crisis and in routine practice: occupational health and psychological support services; availability of materials and equipment for quality clinical practice; volume and workflow adapted to the complexity of clinical practice, performance criteria freed from a “business” logic; policy that prevents excesses in terms of holiday deprivation and imposed overtime; organizational governance and leadership that reinforce collaboration, trust and transparent communication between professionals and hierarchy.
- **Ensuring better drugs and medical equipment production capacities:** the threat of shortages of drugs and medical equipment have put forward the great dependence of Quebec and Canada on external suppliers. There is a need for a strategic national stockpile, and the strengthening of the local industry capacities for the production of essential drugs and medical equipment. Better cooperation and coordination between the federal government and the other provinces is important to develop this industry, particularly with better synergy and mutualization of efforts (133, 140). There is also a need to improve logistics infrastructures for better supply chain management (136).
- **Promoting pluralistic governance and adaptive leadership:** the crisis has shown that governance is much more than a technocratic exercise. Beyond a “romantic and heroic” view of unitary leadership, it is important to ensure the conditions for a plural, agile and adaptive leadership capable of mobilizing a diversity of stakeholders, especially when it comes to making decisions on an evidence-based or emerging science, or ideological basis (141). Such leadership has to cope with divergent visions and interests at different levels of governance (e.g., federal, provincial, municipal, knowledge-based agencies), and transcend sectoral, professional, disciplinary, socio-political as well as ideological or symbolic boundaries (142, 143). A climate of trust, mutual respect, and appropriate space to make explicit and clarify emerging conflicts and divergent views are more likely to generate multiple solutions and capacities to intervene effectively (144).
- **Strengthening independence and agility of knowledge-based agencies:** the crisis has affirmed the central place of knowledge-based agencies in the production of evidence and decision-making support, sometimes under very difficult conditions. Though governments are expected to make decisions based on the best available evidence and practices, tensions between political (the need to act quickly), economic (the need to produce and consume) and scientific (the need to conduct rigorous research) dimensions inevitably arise in uncertain, complex and rapidly changing emergency situations (145–147). In situations of uncertainty and/or conflict, certain stakeholders may have defensive reactions and sometimes refuse to share data (148). The need for independent and agile agencies that have sufficient autonomy and nurture a critical perspective is key to rigorous, transparent, and verifiable decision-making (147, 149–151): “*truth is neither liberal nor conservative*” (152).
- **Improving communication and trust in government and institutions:** communication has emerged as a central element in crisis management, especially in its role in building, maintaining and/or weakening trust in government and institutions in times of crisis. With social networks and digital media, the classic mechanisms of communication, management, and decision-making quickly showed their limits. A high degree of transparency and involvement of the population public affairs should be encouraged and promoted. Spaces for dialogue and exchange, protected by a clear legal framework, are essential to establish audible communication that is accepted as legitimate and necessary for trust in democratic institutions and within society. For example, given the exceptional power that the government has in a such context, it would be relevant to create “wise people committees,” with representatives of citizens and communities, especially to continue to reflect and address the rights of persons and the population in times of crisis (131, 153).

## Implications For The Future and Conclusion

The COVID-19 pandemic highlighted the extreme complexity that characterizes the management of systemic and global health crises. This paper provides a structured holistic analysis of the Quebec HSSS preparedness to manage health crises of magnitude, as well as the choices and strategies adopted during the COVID-19 crisis. Using the framework of Palagyi et al. (28), we shed light on the empirical and practical complexity of decision-making and action in a health emergency context, examining more specifically: (1) how surveillance was structured; (2) how workforce issues were managed; (3) what infrastructures and medical supplies were made available; (4) what communication mechanisms were put in place; (5) what form of governance emerged; and (6) whether trust was established and maintained throughout the crisis (28). Such a holistic analysis looked at the evolution of the first wave of the COVID-19 pandemic and at the Quebec HSSS's capacity to adapt and deploy effective interdependent measures to face the crisis.

The implications of our findings are in line with other studies on the preparedness of health systems against outbreaks of infectious diseases and natural hazards (2, 9, 11, 28, 47, 57, 124, 132, 135, 149, 154, 155). The key ingredients for managing sanitary crises have led the HSSS to: (1) maintain basic essential health services; (2) remove barriers to access emergency care and services; (3) provide rapid and flexible access to required financial resources; (4) deploy leadership through a clear and flexible chain of command; (5) ensure optimal collaboration and coordination among stakeholders inside and outside the HSSS; (6) modify and adapt standards and protocols of care; (7) nurture a skilled, trained and ready-to-act workforce; (8) obtain medical supplies and equipment; (9) implement protocols and train health care providers in infection prevention and control; and (10) strengthen post-crisis recovery plans (156). In sum, COVID-19 has shown that HSSS are resilient when they are already strong (28, 156). It reminds us that emergency preparedness, rather than reaction, is an imperative investment (131).

Horton (157) qualified COVID-19 as a “syndemic” because it challenges society as a whole (e.g., health and social policies, socio-economic inequalities, functioning of democratic institutions, environment) (157). Syndemics reveal the potential for systemic transformation and profound changes (47, 157). This crisis is a moment of truth for HSSS and societies (158). Though it revealed systemic weaknesses and mistakes motivated by fear and anxiety, COVID-19 also generated learnings and opportunities for necessary disruptive innovations and transformations at all levels (158). Thus, rather than a failure, the COVID-19 crisis should be considered as an opportunity to sustainably improve the functioning of HSSS and societies (47, 157). It is therefore necessary to leave aside a narrow crisis management approach and seek to better understand the complexity of the dynamics and interactions involved (157).

This paper is not without limitations. In the absence of comprehensive empirical data, it is impossible to draw assertive conclusions. Nonetheless, our analyses shed light on lessons learned and can inform decisions about future research and empirical evaluations. Ultimately, it would be extremely useful to identify the capacities and conditions that are essential for HSSS to be able to prevent, detect and respond in a timely and effective manner to future health crises. According to Jacobsen (136), “(…) *the world does not need more evidence of the health, social, economic, environmental, and other problems that arise when we fail to invest adequately in global health security. What is required to break this panic-then-forget cycle is to follow through on prioritizing, funding, and implementing preparedness interventions*” (136). The COVID-19 crisis has reminded us how interdependent and interconnected our societies are. The HSSS operate within a global ecosystem where choices made elsewhere can have a major impact on the other side of the planet, and vice versa. Some countries have been more affected than others and some countries have coped with the crisis better than others. In this regard, there is a need to conduct a comparative research and analyses integrated into mutual learning processes, as well as genuine policy discussions on the management of the COVID-19 crisis in different countries. Such global comparisons and open dialogue could inform decision-making for actions and policies that can improve our HSSS and societies. As Bashshur et al. (18) stress, “*the actual benefits would accrue only if the correct lessons are drawn from the difficult experience just passed, and there is a political will to embrace genuine reform. The current situation with the coronavirus (COVID-19) pandemic is no exception*” (18).

## Author Contributions

HA, PL, RF, J-PF, JL, RA, SC, MAS, MS, and MA were engaged in the drafting of the manuscript and they all read and approved the final manuscript.

## Conflict of Interest

The authors declare that the research was conducted in the absence of any commercial or financial relationships that could be construed as a potential conflict of interest.

## References

[1] Beveridge W. Social Insurance and Allied Services. London: Her Majesty's Stationery Office (1942).

[2] Capano G. Policy design and state capacity in the COVID-19 emergency in Italy: if you are not prepared for the (un) expected, you can be only what you already are. Policy Soc. (2020) 39:326–44. 10.1080/14494035.2020.1783790 PMC8754691 35039724

[3] Gates B. Responding to Covid-19—a once-in-a-century pandemic? N Engl J Med. (2020) 382:1677–9. 10.1056/NEJMp2003762 32109012

[4] Latifi R Doarn CR. Perspective on COVID-19: finally, telemedicine at center stage. Telemed e-Health. (2020) 26:1106–9. 10.1089/tmj.2020.0132 32408804

[5] Parmet WE Sinha MS. Covid-19—the law and limits of quarantine. N Engl J Med. (2020) 382:e28. 10.1056/NEJMp2004211 32187460

[6] McAleer M. Prevention is better than the cure: risk management of COVID-19. J Risk Financial Manage. (2020) 13:46. 10.3390/jrfm13030046

[7] Yue X-G Shao X-F Li RYM Crabbe MJC Mi L Hu S. Risk management analysis for novel coronavirus in Wuhan, China. J Risk Financial Manage. (2020) 13:1–6. 10.3390/jrfm13020022

[8] Gatto M Bertuzzo E Mari L Miccoli S Carraro L Casagrandi R. Spread and dynamics of the COVID-19 epidemic in Italy: Effects of emergency containment measures. Proc Natl Acad Sci USA. (2020) 117:10484–91. 10.1073/pnas.2004978117 32327608 PMC7229754

[9] Hsiang S Allen D Annan-Phan S Bell K Bolliger I Chong T. The effect of large-scale anti-contagion policies on the COVID-19 pandemic. Nature. (2020) 584:262–7. 10.1038/s41586-020-2404-8 32512578

[10] Loayza NV Pennings S. Macroeconomic policy in the time of COVID-19: a primer for developing countries. World Bank. (2020) 28:1–9. 10.1596/33540

[11] Migone AR. Trust, but customize: federalism's impact on the Canadian COVID-19 response. Policy Soc. (2020) 39:382–402. 10.1080/14494035.2020.1783788 PMC8754695 35039727

[12] Dzigbede K Gehl SB Willoughby K. Disaster resiliency of US local governments: insights to strengthen local response and recovery from the COVID-19 pandemic. Public Admin Rev. (2020) 80:634–43. 10.1111/puar.13249 PMC7300832 32836460

[13] Raamkumar AS Tan SG Wee HL. Measuring the outreach efforts of public health authorities and the public response on facebook during the COVID-19 pandemic in early 2020: cross-country comparison. J Med Internet Res. (2020) 22:e19334. 10.2196/19334 32401219 PMC7238862

[14] Director General. WHO Director-General's Introductory Remarks for the Launch of the GPMB 2020 Annual Report: A World in Disorder. World Health Organization (2020). Available online at: https://www.who.int/director-general/speeches/detail/who-director-general-s-introductory-remarks-for-the-launch-of-the-gpmb-2020-annual-report-a-world-in-disorder (accessed February 12, 2021).

[15] Haldane V Ong SE Chuah FLH Legido-Quigley H. Health systems resilience: meaningful construct or catchphrase? Lancet. (2017) 389:1513. 10.1016/S0140-6736(17)30946-7 28422019 PMC7133569

[16] Hanvoravongchai P Mounier-Jack S Oliveira Cruz V Balabanova D Biellik R Kitaw Y. Impact of measles elimination activities on immunization services and health systems: findings from six countries. J Infect Dis. (2011) 204:S82–9. 10.1093/infdis/jir091 21666218

[17] Mounier-Jack S Griffiths UK Closser S Burchett H Marchal B. Measuring the health systems impact of disease control programmes: a critical reflection on the WHO building blocks framework. BMC Public Health. (2014) 14:278. 10.1186/1471-2458-14-278 24666579 PMC3974593

[18] Bashshur RL Doarn CR Frenk JM Kvedar JC Shannon GW Woolliscroft JO. Beyond the COVID pandemic, telemedicine, and health care. Telemed e-Health. (2020) 26:1310–3. 10.1089/tmj.2020.0328 32809913

[19] Institut national de santé publique du Québec. Données COVID-19 au Québec. (2020). Available online at: https://www.inspq.qc.ca/covid-19/donnees (accessed February 12, 2021).

[20] Western-University. COVID-19 Canada. Western-University (2020). Available online at: https://covid-19-canada.uwo.ca/en/data.html (accessed February 12, 2021).

[21] COVID-19 Canada. Ressources sur la COVID-19. Esri Canada (2020). Available online at: https://ressouces-fr-covid19canada.hub.arcgis.com/?locale=fr (accessed February 12, 2021).

[22] Government of Ontario. Données sur les cas de COVID-19: Les foyers de soins de longue durée. Government of Ontario (2020). Available online at: https://covid-19.ontario.ca/fr/data/les-foyers-de-soins-de-longue-duree (accessed February 12, 2021).

[23] Government of Ontario. Données sur les cas de COVID-19: l'Ontario dans son ensemble. Government of Ontario (2020). Available online at: https://covid-19.ontario.ca/fr/data#testing-data-reporting-sources (accessed February 12, 2021).

[24] Public Health Agency of Canada. Canada COVID-19 Situational Awareness Dashboard. Public Health Agency of Canada (2020). Available online at: https://phac-aspc.maps.arcgis.com/apps/opsdashboard/index.html#/e968bf79f4694b5ab290205e05cfcda6%5c (accessed February 12, 2021).

[25] British Columbia Centre for Disease Control. British Columbia COVID-19 Daily Situation Report (2020). Vancouver, BC.

[26] McElroy J. COVID-19 in British Columbia by the Numbers. CBC (2020). Available online at: https://www.cbc.ca/news/canada/british-columbia/covid-19-british-columbia-charts-1.5510000 (accessed February 12, 2021).

[27] Denis JL Côté N Fleury C Currie G Spyridonidis D. Global health and innovation: a panoramic view on health human resources in the COVID-19 pandemic context. Int J Health Plann Manage. (2021) 202:1–13. 10.1002/hpm.3129 33647168 PMC8014483

[28] Palagyi A Marais BJ Abimbola S Topp SM McBryde ES Negin J. Health system preparedness for emerging infectious diseases: a synthesis of the literature. Glob Public Health. (2019) 14:1847–68. 10.1080/17441692.2019.1614645 31084412

[29] Sheikh K Gilson L Agyepong IA Hanson K Ssengooba F Bennett S. Building the field of health policy and systems research: framing the questions. PLoS Med. (2011) 8:e1001073. 10.1371/journal.pmed.1001073 21857809 PMC3156683

[30] Alvesson M Sandberg J. Generating research questions through problematization. Acad Manage Rev. (2011) 36:247–71. 10.5465/AMR.2011.59330882

[31] Miles MB Huberman AM Saldaña J. Qualitative Data Analysis: A Methods Sourcebook. 3rd. ed: Thousand Oaks, CA: Sage (2014).

[32] Deber R. Treating Health Care: How the Canadian System Works and How It Could Work Better. Treating Health Care: Toronto, ON: University of Toronto Press (2019). 10.3138/9781487513450 30741149

[33] Bakvis H Skogstad G. Canadian Federalism: Performance, Effectiveness, and Legitimacy. Toronto, ON: University of Toronto Press (2020). 10.3138/9781487570460

[34] Gouvernement du Canada. Loi canadienne sur la santé. (1984). Article 7 et suivants: Gouvernement du Canada. Available online at: https://laws-lois.justice.gc.ca/fra/lois/c-6/TexteComplet.html (accessed July 11, 2020).

[35] World Health Organization. Pandemic Influenza Risk Management: A WHO Guide to Inform and Harmonize National and International Pandemic Preparedness and Response. Geneva: World Health Organization (2017).

[36] Gouvernement du Canada. Règlement Sanitaire International - Évaluation Extérieure Conjointe du Canada : Rapport auto-évaluation. (2018). Available online at: https://www.canada.ca/fr/sante-publique/services/mesures-interventions-urgence/reglement-sanitaire-international-evaluation-exterieure-conjointe-canada-rapport-auto-evaluation.html#sec41 (accessed September 2, 2020).

[37] Alami H Fortin JP Gagnon MP Pollender H Têtu B Tanguay F. The challenges of a complex and innovative telehealth project: a qualitative evaluation of the eastern Quebec Telepathology network. Int J Health Policy Manage. (2018) 7:421. 10.15171/ijhpm.2017.106 29764106 PMC5953525

[38] Turgeon J Gagnon F. Le rôle de l'État dans la dispensation des services de santé. L'Observatoire de l'administration publique de l'École nationale d'administration publique (Quebec City, QC). (2006) Report No.: 2923008480.

[39] Ministère de la santé et des services sociaux. Établissements de santé et de services sociaux : Centre d'hébergement et de soins de longue durée. Gouvernement du Québec. Available online at: https://www.msss.gouv.qc.ca/reseau/etablissements-de-sante-et-de-services-sociaux/#chsld (accessed Auguste 25, 2020).

[40] Touzin C Lacoursière A Teisceira-Lessard P Gagnon K. La santé: le mastodonte bureaucratique. La Presse (2020). Available online at: https://www.lapresse.ca/actualites/enquetes/2020-05-02/reseaude-la-sante-le-mastodonte-bureaucratique (accessed July 17, 2020).

[41] Sampson X. Crise dans les CHSLD: une succession de réformes malavisées. Radio-Canada (2020). Available online at: https://ici.radio-canada.ca/nouvelle/1700150/reforme-sante-barrette-couillard-consequences-structure-covid (accessed July 17, 2020).

[42] Fiset-Laniel J Ak'ingabe Guyon RP Strumpf EC. Public health investments: neglect or wilful omission? Historical trends in Quebec and implications for Canada. Canad J Public Health. (2020) 111:383–8. 10.17269/s41997-020-00342-1 32514719 PMC7278767

[43] Archambault H. Deux fois plus de médecins au privé depuis cinq ans. Le Journal de Montréal (2018). Available online at: https://www.journaldemontreal.com/2018/11/16/deux-fois-plus-de-medecins-au-prive-depuis-cinq-ans (accedded September 20, 2020).

[44] Institut national de santé publique du Québec. Mission. Available online at: https://www.inspq.qc.ca/institut/qui-sommes-nous (accessed July 11, 2020).

[45] Gouvernement du Québec. Loi sur l'Institut national d'excellence en santé et en services sociaux. Gouvernement du Québec. Available online at: http://www.legisquebec.gouv.qc.ca/fr/showdoc/cs/i-13.03 (accessed September 15, 2020).

[46] Institut national de santé publique du Québec. Ligne du temps COVID-19 au Québec. Institut national de santé publique (2020). Available online at: https://www.inspq.qc.ca/covid-19/donnees/ligne-du-temps (accessed August 25, 2020).

[47] Willi Y Nischik G Braunschweiger D Pütz M. Responding to the COVID-19 crisis: transformative governance in Switzerland. Tijdschr Econ Soc Geogr. (2020) 111:302–17. 10.1111/tesg.12439 32836490 PMC7323205

[48] Leon DA Shkolnikov VM Smeeth L Magnus P Pechholdová M Jarvis CI. COVID-19: a need for real-time monitoring of weekly excess deaths. Lancet. (2020) 395:e81. 10.1016/S0140-6736(20)30933-8 32333839 PMC7176374

[49] The Economist. Tracking Covid-19 Excess Deaths Across Countries. The Economist (2020). Available online at: https://cutt.ly/9lcU8RQ (accessed September 16, 2020).

[50] Gerbet T. Un bilan bien plus lourd qu'annoncé à l'Institut universitaire de gériatrie de Montréal. Radio-Canada (2020). Available online at: https://ici.radio-canada.ca/nouvelle/1693690/hecatombe-institut-geriatrie-montreal-deces-covid-chsld (accessed July 25, 2020).

[51] Pilon-Larose H Marquis M. La fin des décomptes par ≪fax≫, annonce Legault. La Presse (2020). Available online at: https://www.lapresse.ca/covid-19/2020-06-05/la-fin-des-decomptes-par-fax-annonce-legault (accessed July 17, 2020).

[52] Estabrooks CA Straus SE Flood CM Keefe J Armstrong P Donner GJ. Restoring trust: COVID-19 and the future of long-term care in Canada. FACETS. (2020) 5:651–91. 10.1139/facets-2020-0056

[53] Doucet H. ≪Répondre à la vulnérabilité≫: l'éthique et les CHSLD au temps de la Covid-19. É*thique Santé*. (2020) 17:142–6. 10.1016/j.etiqe.2020.07.003 PMC7373036 32837545

[54] Béland D Marier P. COVOID-19 and long-term care policy for older people in Canada. J Aging Soc Policy. (2020) 32:358–64. 10.1080/08959420.2020.1764319 32419658

[55] Vérificateur général du Québec. Personnes âgées en perte d'autonomie : Services d'hébergement. Rapport du Vérificateur général du Québec à l'Assemblée nationale du Québec (Quebec City, QC) (2012).

[56] Michaud PC. Soins de longue durée: une opportunité de réflexion pour ne plus répéter les mêmes erreurs. CIRANO (Montreal, QC) (2020).

[57] Denis JL Potvin L Rochon J Fournier P Gauvin L. On redesigning public health in Québec: lessons learned from the pandemic. Canad J Public Health. (2020) 111:912–20. 10.17269/s41997-020-00419-x 33063289 PMC7561231

[58] World Health Organization. COVID-19 Virtual Press Conference Geneva: World Health Organization (2020).

[59] Hancock T Kirk M MacDonald M Neudorf C Sutcliffe P Talbot J. The weakening of public health: a threat to population health and health care system sustainability. Canad J Public Health. (2017) 108:e1–6. 10.17269/CJPH.108.6143 28425892 PMC6972173

[60] Naylor C. Leçons de la crise du SRAS: renouvellement de la santé publique au Canada. Comité consultatif national sur le SRAS et la santé publique (Ottawa, ON) (2003).

[61] Lemay F Vanderschuren A Alain J. Aeromedical evacuations during the COVID-19 pandemic: practical considerations for patient transport. Canad J Emerg Med. (2020) 22:584–6. 10.1017/cem.2020.434 32576326 PMC7364053

[62] Fleet R. COVID-19 : les régions rurales sont-elles dans notre angle mort? Le Soleil (2020). Available online at: https://www.lesoleil.com/opinions/point-de-vue/covid-19–les-regions-rurales-sont-elles-dans-notre-angle-mort-377497f95aea33d7072d992e04f65f2f (accessed September 4, 2020).

[63] Arsenault M Evans B Karanofsky M Gardie J Schulha M. Covid-19–Exercer la télémédecine durant la pandémie. Canad Family Phys. (2020). Available online at: https://www.cfp.ca/news/2020/03/26/3-26-2 (accessed September 25, 2020).

[64] Breton M Deville-Stoetzel N Motulsky A Lussier MT Layani G Gaboury I. Portrait de la transformation rapide vers les téléconsultations pendant la COVID-19 dans les Groupes de médecines familiales-Universitaires. Réseau québecois COVID pandémie (2020). Available online at: https://rqcp.ca/wpcontent/uploads/2020/11/Portrait-teleconsultations_GMFU_30octobre_2020.pdf (accessed December 27, 2020).

[65] Alami H Gagnon MP Fortin JP. Some multidimensional unintended consequences of telehealth utilization: a multi-project evaluation synthesis. Int J Health Policy Manage. (2019) 8:337. 10.15171/ijhpm.2019.12 31256566 PMC6600023

[66] Alami H Lamothe L Fortin JP Gagnon MP. L'implantation de la télésanté et la pérennité de son utilisation au Canada: quelques leçons à retenir. La Recherche Europ Téléméd. (2016) 5:105–17. 10.1016/j.eurtel.2016.10.001

[67] Gouvernement du Québec. Arrêté numéro 2020-026 de la ministre de la Santé et des Services sociaux en date du 20 avril (Quebec City, QC) (2020).

[68] Beauchet O. The expert COVID-19 team for older persons of the Quebec Health and Social Services Ministry. Aging Clin Exp Res. (2020) 32:1625–26. 10.1007/s40520-020-01623-y 32548742 PMC7297135

[69] Holroyd-Leduc JM. Continuing care and COVID-19: a Canadian tragedy that must not be allowed to happen again. Canad Med Assoc J. (2020) 192:E632–3. 10.1503/cmaj.201017 32409521 PMC7867213

[70] Da Silva RB Hébert R Blais R. Repenser l'allocation des ressources humaines en santé: faut-il vraiment faire un choix entre CHSLD et hôpitaux? Montreal, QC: CIRANO (2020).

[71] Laliberté-Auger F Côté-Sergent A Décarie Y Duclos JY Michaud P-C. Utilisation et coût de l'hébergement avec soins de longue durée au Québec, 2010 à 2050. Montreal, QC: CIRANO (2015).

[72] Dubois CA. COVID-19 et main-d'œuvre en santé: des politiques de rupture pour briser le cycle de reproduction des fragilités actuelles. Montreal, QC: CIRANO (2020).

[73] Canadian Medical Association. Statement From CMA President on the Impact of COVID-19 on Health Workers in Canada. Canadian-Medical-Association (2020). Available online at: https://www.newswire.ca/news-releases/statement-from-cma-president-on-the-impact-of-covid-19-on-health-workers-in-canada-823758203.html (accessed September 3, 2020).

[74] Institut national d'excellence en santé et services sociaux. COVID-19 et la détresse psychologique et la santé mentale du personnel du réseau de la santé et des services sociaux dans le contexte de l'actuelle pandémie. Montreal, QC: Institut national d'excellence en santé et services sociaux (2020).

[75] McMahon M Nadigel J Thompson E Glazier RH. Informing Canada's health system response to COVID-19: priorities for health services and policy research. Healthcare Policy. (2020) 16:112. 10.12927/hcpol.2020.26249 32813643 PMC7435075

[76] Institut national d'excellence en santé et services sociaux. COVID-19: Pandémie et travailleurs de la santé. Institut national de santé publique du Québec (2020).

[77] Carrière G Park J Deng Z Kohen D. Heures supplémentaires travaillées par le personnel professionnel en soins infirmiers pendant la pandémie de COVID-19. Ottawa, ON: Statistique Canada (2020).

[78] Radio-Canada. Plus de 10 000 employés absents dans le réseau de la santé au Québec. Radio-Canada (2020). Available online at: https://ici.radio-canada.ca/nouvelle/1698334/covid-19-coronavirus-employes-absents-reseau-sante-quebec (accessed July 17, 2020).

[79] Porter I. Des cadres en santé se disent lésés d'être sans prime pendant la crise. Le Devoir (2020). Available online at: https://www.ledevoir.com/societe/sante/580394/coronavirus-des-cadres-en-sante-se-disent-leses-d-etre-sans-prime (accessed July 17, 2020).

[80] Beaulieu S Couillard M. Avis scientifique sur le port du masque dans la communauté en situation de pandémie d'influenza. Montreal, QC: Institut national de santé publique du Québec (2007).

[81] Institut national de santé publique du Québec. Réutilisation des respirateurs N95 dans un contexte d'une pénurie réelle ou appréhendée lors de la pandémie de la COVID-19. Montreal, QC: Institut national de santé publique du Québec (2020).

[82] Perron L. 45 millions pour des masques qui n'existent pas. La Presse (2020). Available online at: https://www.lapresse.ca/covid-19/2020-04-30/45-millions-pour-des-masques-qui-n-existent-pas (accessed July 19, 2020).

[83] Saba R. Pharmacies Having Trouble Keeping up as Refill Requests Surge: The Star (2020). Available online at: https://www.thestar.com/business/2020/03/19/please-stop-stockpiling-prescriptions-pharmacies-urge-canadians.html (accessed July 11, 2020).

[84] Santé Canada. Pénurie d'inhalateurs de salbutamol au Canada. Gouvernement du Canada (2020). Available online at: https://canadiensensante.gc.ca/recall-alert-rappel-avis/hc-sc/2020/72833a-fra.php (accessed August 25, 2020).

[85] Cassels A. Most of our prescription drugs are manufactured overseas—but are they safe? Canad Med Assoc J. (2012) 184:1648. 10.1503/cmaj.120416 22966061 PMC3470633

[86] Eggertson L. Canada can override patents to combat drug, equipment shortages during the pandemic. CMAJ. (2020) 192:E438. 10.1503/cmaj.1095862 32312830 PMC7207185

[87] Bowles SK. Drug shortages: more than just background noise. Canad J Hosp Pharmacy. (2019) 72:3–4. 10.4212/cjhp.v72i1.2860 30828087 PMC6391244

[88] Rinaldi F de Denus S Nguyen A Nattel S Bussières J-F. Drug shortages: patients and health care providers are all drawing the short straw. Canad J Cardiol. (2017) 33:283–6. 10.1016/j.cjca.2016.08.010 27923583

[89] Barro K Malone A Mokede A Chevance C. Gestion de l'épidémie de la COVID-19 par les établissements publics de santé–Analyse de la Fédération Hospitalière de France. J De Chirurgie Viscerale. (2020) 157:520–4. 10.1016/j.jchirv.2020.04.009 PMC7165272 32313587

[90] Feral-Pierssens AL Claret PG Chouihed T. Collateral damage of the COVID-19 outbreak: expression of concern. Europ J Emerg Med. (2020) 27:233–4. 10.1097/MEJ.0000000000000717 32345850 PMC7202126

[91] Gerbet T. ≪Bombes à retardement≫ : des victimes d'infarctus et d'AVC évitent les urgences. Radio-Canada (2020). Available online at: https://ici.radio-canada.ca/nouvelle/1693406/coronavirus-crise-cardiaque-urgence-covid-infarctus-avc (accessed July 11, 2020).

[92] Institut national d'excellence en santé et services sociaux. COVID-19 et présentation clinique d'infarctus. Montreal, QC: Institut national d'excellence en santé et services sociaux (2020).

[93] Gerbet T. Déjà des personnes décédées indirectement de la COVID-19. Radio-Canada (2020). Available online at: https://ici.radio-canada.ca/nouvelle/1695697/deces-cardiaque-urgences-hopitaux-medecins-quebec-covid (accessed July 11, 2020).

[94] Marchand C. Hausse inquiétante des complications liées aux infarctus. Radio-Canada (2020). Available online at: https://ici.radio-canada.ca/nouvelle/1695566/coronavirus-infarctus-complications-coeur-crise-cardiaque-quebec (accessed August 25, 2020).

[95] Jacques F Voisine P Perrault L. Cardiovascular Collateral damages at the time of COVID-19. Canad J Cardiol. (2020) 36:1327. e7. 10.1016/j.cjca.2020.06.011 32585328 PMC7308022

[96] La Presse canadienne. Sondage : la pandémie de COVID-19 nuit aux soins en oncologie au Québec. Radio-Canada (2020). Available online at: https://ici.radio-canada.ca/nouvelle/1694452/patients-cancer-affectes-effets-gestion-crise-covid-19 (accessed July 11, 2020).

[97] Duchaine H. 4000 cancers non diagnostiqués. Le Journal de Québec (2021). Available online at: https://www.journaldequebec.com/2021/01/22/4000-cancers-non-diagnostiques (accessed February 11, 2021).

[98] Radio-Canada. Les retards s'accumulent dans le traitement du cancer au Québec. Radio-Canada (2020). Available online at: https://ici.radio-canada.ca/nouvelle/1702143/retards-traitement-cancer-quebec (accessed July 17, 2020).

[99] Radio-Canada. Reprise des chirurgies non urgentes : de l'espoir, mais d'énormes retards à rattraper. Radio-Canada (2020). Available online at: https://ici.radio-canada.ca/nouvelle/1704425/reprise-chirurgies-non-urgentes-espoir-retards-cancer- (accessed July 17, 2020).

[100] Jayasinha R Nairn S Conrod P. A dangerous “Cocktail”: the COVID-19 pandemic and the youth opioid crisis in North America: a response to vigo et al. Canad J Psychiatry. (2020) 65:692–4. 10.1177/0706743720943820 32701377 PMC7502881

[101] Institut national de santé publique du Québec. COVID-19: Pandémie, bien-être et santé mentale. Montreal, QC: Institut national de santé publique du Québec (2020).

[102] Institut national de santé publique du Québec. Réponse rapide : COVID-19 et répercussions psychosociales. Montreal, QC: Institut national de santé publique du Québec (2020).

[103] Emanuel EJ Persad G Upshur R Thome B Parker M Glickman A. Fair allocation of scarce medical resources in the time of Covid-19. N Engl J Med. (2020) 382:2049–55. 10.1056/NEJMsb2005114 32202722

[104] Potvin L. Public health saves lives: sad lessons from COVID-19. Canad J Public Health. (2020) 111:308–11. 10.17269/s41997-020-00344-z 32483649 PMC7263179

[105] Raynault M Lacroix A Quirion R. Approche québécoise de Santé publique face à la pandémie de COVID-19. Bull De L'Acad Nationale De Med. (2020) 204:741–3. 10.1016/j.banm.2020.06.009 PMC7280803 32834055

[106] Merkley E Bridgman A Loewen PJ Owen T Ruths D Zhilin O. A rare moment of cross-partisan consensus: elite and public response to the COVID-19 Pandemic in Canada. Canad J Political Sci. (2020) 53:311–18. 10.1017/S0008423920000311

[107] Crête M. Le masque superflu, puis essentiel. Le Devoir (2020). Available online at: https://www.ledevoir.com/societe/583180/superflu-puis-essentiel (accessed September 2, (2020).

[108] Collectif. Pour un ≪masque≫ non médical pour tous. Le Devoir (2020). Available online at: https://www.ledevoir.com/opinion/idees/576603/pour-un-masque-non-medical-pour-tous (accessed September 2, 2020).

[109] Lachance N. La COVID-19 inquiète les médecins de famille: ils demandent des mesures drastiques et la télémédecine. Le Journal de Québec (2020). Available online at: https://www.journaldequebec.com/2020/03/12/la-covid-19-inquiete-les-medecins-de-famille-ils-demandent-des-mesurent-drastiques (accessed May 2, 2020).

[110] Teichmann L Nossek S Bridgman A Loewen P Owen T Ruths D. Public health communication and engagement on social media during the COVID-19 pandemic. OSF Preprints. (2020) 1–41. 10.2196/preprints.23248

[111] Bridgman A Merkley E Loewen PJ Owen T Ruths D Teichmann L. The causes and consequences of covid-19 misperceptions: understanding the role of news and social media. Harvard Kennedy Sch Misinformat Rev. (2020) 1:1–18. 10.37016/mr-2020-028

[112] Yates J. COVID-19: comment les conspirations à propos de la 5G ont envahi l'actualité. Radio-Canada (2020). Available online at: https://ici.radio-canada.ca/nouvelle/1700255/covid-5g-conspiration-pourquoi-complot-yonder-tour-sante-effet (accessed July 11, (2020).

[113] Radio-Canada. COVID-19: le Québec s'adresse aux jeunes, l'Ontario peine à le faire. Radio-Canada (2020). Available online at: https://ici.radio-canada.ca/nouvelle/1722222/covid19-jeunes-communication-ontario-quebec-legault-ford (accessed September 16, (2020).

[114] Gouvernement du Canada. Plan d'intervention économique du Canada pour répondre à la COVID-19. (2020). Available online at: https://www.canada.ca/fr/ministere-finances/plan-intervention-economique.html (accessed August 25, 2020).

[115] Bell M. Premiers don't want Emergencies Act Used During COVID-19 Pandemic. The Globe and Mail (2020). Available online at: https://www.theglobeandmail.com/canada/article-premiers-dont-want-emergencies-act-used-during-covid-19-pandemic-2/ (accessed April 15, 2020).

[116] Adeel AB Catalano M Catalano O Gibson G Muftuoglu E Riggs T. COVID-19 policy response and the rise of the sub-national governments. Canad Public Policy. (2020) 46:565–84. 10.3138/cpp.2020-101 PMC9400820 36039151

[117] Radio-Canada. Le Québec franchit la barre des 23 000 cas de COVID-19. Radio-Canada (2020). Available online at: https://ici.radio-canada.ca/nouvelle/1697419/coronavirus-bilan-quebec-statistiques-25-avril (accessed May 25, 2020).

[118] Institut national d'excellence en santé et services sociaux. COVID-19 et application mobile de télésuivi de signes vitaux. Montreal, QC: Institut national d'excellence en santé et services sociaux (2020).

[119] Institut national de santé publique du Québec. Recommandations Intérimaires COVID-19: port du couvre-visage ou du masque médical par la population générale. Montreal, QC: Institut national de santé publique du Québec (2020).

[120] Gerbet T. Écoles: Québec n'a pas suivi l'avis de l'Institut national de santé publique. Radio-Canada (2020). Available online at: https://ici.radio-canada.ca/nouvelle/1740622/ministere-education-avis-sante-publique-taille-classe-nombre-eleves (accessed December 15, 2020).

[121] Schué R Boily D. Des recommandations de la santé publique n'ont pas été suivies par le gouvernement Legault. Radio-Canada (2020). Available online at: https://ici.radio-canada.ca/nouvelle/1722826/covid-isolement-symptomes-courriels-legault-arruda-drouin-montreal-quebec (accessed September 8, 2020).

[122] Gerbet T. Les rares avis écrits du Dr Arruda resteront secrets. Radio-Canada (2021). Available online at: https://ici.radio-canada.ca/nouvelle/1762439/avis-ecrits-arruda-secrets-quebec-legault-covid (accessed February 12, 2021).

[123] Alwan NA Bhopal R Burgess RA Colburn T Cuevas LE Smith GD. Evidence informing the UK's COVID-19 public health response must be transparent. Lancet. (2020) 395:1036–7. 10.1016/S0140-6736(20)30667-X 32197104 PMC7270644

[124] Kruk ME Myers M Varpilah ST Dahn BT. What is a resilient health system? Lessons from Ebola. Lancet. (2015) 385:1910–2. 10.1016/S0140-6736(15)60755-3 25987159

[125] Girard J. Des ≪lanceurs d'alerte≫ se taisent par peur des représailles. Radio-Canada (2020). Available online at: https://ici.radio-canada.ca/nouvelle/1699246/coronavirus-sante-chsld-hopitaux-danielle-mccann-opposition-quebec (accessed July 17, 2020).

[126] Ministre de la santé et des services sociaux. Pandémie de la COVID-19 - On vous écoute Gouvernement du Québec. (2020). Available online at: https://www.msss.gouv.qc.ca/ministere/salle-de-presse/communique-2114/ (accessed July 17, 2020).

[127] Chouinard T. ≪Fin de l'omerta≫ en santé: 1791 dénonciations reçues. La Presse (2020). Available online at: https://www.lapresse.ca/covid-19/2020-05-22/fin-de-l-omerta-en-sante-1791-denonciations-recues (accessed July 17, 2020).

[128] Observatoire international sur les impacts sociétaux de l'IA et du numérique. Analyse sur l'application de notification de contacts COVID-19. Observatoire international sur les impacts sociétaux de l'IA et du numérique (OBVIA) (Montreal, QC) (2020).

[129] Gouvernement du Québec. COVID-19 : une application mobile. Le gouvernement veut votre avis!. Gouvernement du Québec (2020). Available online at: https://consultation.quebec.ca/processes/covidapp (accessed September 16, 2020).

[130] Lehman JG. A Brief Explanation of The Overton Window. Mackinac Center for Public Policy (2010). Available online at: https://www.mackinac.org/OvertonWindow (accessed February 2, 2021).

[131] Régis C Denis JL Gaudreault-DesBiens J-F. Réfléchir à l'après-crise en politique et en santé. Options politiques (2020). Available online at: https://policyoptions.irpp.org/fr/magazines/may-2020/reflechir-a-lapres-crise-en-politique-et-en-sante/ (accessed September 21, 2020).

[132] Collins A Florin M-V Renn O. COVID-19 risk governance: drivers, responses and lessons to be learned. J Risk Res. (2020) 23:1073–82. 10.1080/13669877.2020.1760332

[133] Dudoit A. COVID-19: Les six premiers mois - L'urgence d'un nouveau modèle de gouvernance et d'opérations. Montreal, QC: CIRANO (2020).

[134] Morain SR Kass NE Grossmann C. What allows a health care system to become a learning health care system: results from interviews with health system leaders. Learn Health Syst. (2017) 1:e10015. 10.1002/lrh2.10015 31245552 PMC6516720

[135] Bardosh KL de Vries DH Abramowitz S Thorlie A Cremers L Kinsman J. Integrating the social sciences in epidemic preparedness and response: a strategic framework to strengthen capacities and improve Global Health security. Global Health. (2020) 16:1–18. 10.1186/s12992-020-00652-6 33380341 PMC7772799

[136] Jacobsen KH. Will COVID-19 generate global preparedness? Lancet. (2020) 395:1013–4. 10.1016/S0140-6736(20)30559-6 32199074 PMC7270962

[137] Zumla A Dar O Kock R Muturi M Ntoumi F Kaleebu P. Taking forward a 'One Health' approach for turning the tide against the Middle East respiratory syndrome coronavirus and other zoonotic pathogens with epidemic potential. Int J Infect Dis. (2016) 100:5–9. 10.1016/j.ijid.2016.06.012 27321961 PMC7128966

[138] Bodenheimer T Sinsky C. From triple to quadruple aim: care of the patient requires care of the provider. Ann Family Med. (2014) 12:573–6. 10.1370/afm.1713 25384822 PMC4226781

[139] Frush K Lee G Wald SH Hawn M Krna C Holubar M. Navigating the Covid-19 pandemic by caring for our health care workforce as they care for our patients. NEJM Catal Innovat Care Delivery. (2020) 2:1–34. 10.1056/CAT.20.0378

[140] Rousseau H-P. COVID-19 – Idées de politiques économiques de gestion et de sortie de crise pour le Québec et le Canada. CIRANO (2020).

[141] Denis JL Langley A Sergi V. Leadership in the plural. Acad Manage Ann. (2012) 6:211–83. 10.5465/19416520.2012.667612

[142] Ospina SM Foldy EG Fairhurst GT Jackson B. Collective dimensions of leadership: connecting theory and method. Hum Relat. (2020) 73:441–63. 10.1177/0018726719899714

[143] Denis JL Côté N Régis C. Leadership en contexte de pandémie : quelles leçons tirées pour les soins et les services en première ligne? (2020). Available online at: http://reseau1quebec.ca/nouveautes/covid-19/#R%C3%A9flexions (accessed September 3, (2020).

[144] Rutter H Wolpert M Greenhalgh T. Managing uncertainty in the covid-19 era. Br Med J. (2020) 370:m3349. 10.1136/bmj.m3349 32873549

[145] Djulbegovic B Guyatt G. Evidence-based medicine in times of crisis. J Clin Epidemiol. (2020) 126:164–6. 10.1016/j.jclinepi.2020.07.002 32659364 PMC7348606

[146] Weible CM Nohrstedt D Cairney P Carter DP Crow DA Durnová AP. COVID-19 and the policy sciences: initial reactions and perspectives. Policy Sci. (2020) 53:225–41. 10.1007/s11077-020-09381-4 32313308 PMC7165254

[147] Berger L Berger N Bosetti V Gilboa I Hansen LP Jarvis C. Uncertainty and Decision-Making During a Crisis: How to Make Policy Decisions in the COVID-19 Context? University of Chicago, Becker Friedman Institute for Economics Working Paper (Chicago, IL). (2020) 10.2139/ssrn.3647188

[148] Levesque JF Sutherland K Watson DE Currow DC Bolevich Z Koff E. Learning systems in times of crisis: the Covid-19 critical intelligence unit in New South Wales, Australia. NEJM Catalyst Innovat Care Deliv. (2020) 1:1–11. 10.1056/CAT.20.0542

[149] Duffield A Reid G Shoham J Walker D. Evidence base for interventions in complex emergencies. Lancet. (2005) 365:842–3. 10.1016/S0140-6736(05)71034-5 15752520

[150] Blanchet K Ramesh A Frison S Warren E Hossain M Smith J. Evidence on public health interventions in humanitarian crises. Lancet. (2017) 390:2287–96. 10.1016/S0140-6736(16)30768-1 28602563

[151] Brownson RC Fielding JE Green LW. Building capacity for evidence-based public health: reconciling the pulls of practice and the push of research. Ann Rev Public Health. (2018) 39:27–53. 10.1146/annurev-publhealth-040617-014746 29166243 PMC5972383

[152] The Editors. Dying in a leadership vacuum. N Engl J Med. (2020). 383:1479–80. 10.1056/NEJMe2029812 33027574

[153] Béland G. Le virus liberticide. La Presse (2020). Available online at: https://www.lapresse.ca/covid-19/2020-04-19/le-virus-liberticide (accessed September 3, 2020).

[154] Therrien M Normandin J Denis J. Bridging complexity theory and resilience to develop surge capacity in health systems. J Health Organiz Manage. (2017) 31:96. 10.1108/JHOM-04-2016-0067 28260411

[155] Boin A Comfort L Demchak C. The rise of resilience. In: Comfort L Boin A Demchak C, editors. Designing Resilience: Preparing for Extreme Events. Pittsburgh, PA: University of Pittsburgh Press (2020). p. 1–12. 10.2307/j.ctt5hjq0c.5

[156] Nuzzo JB Meyer D Snyder M Ravi SJ Lapascu A Souleles J. What makes health systems resilient against infectious disease outbreaks and natural hazards? Results from a scoping review. BMC Public Health. (2019) 19:1310. 10.1186/s12889-019-7707-z 31623594 PMC6798426

[157] Horton R. Offline: COVID-19 is not a pandemic. Lancet. (2020) 396:874. 10.1016/S0140-6736(20)32000-6 32979964 PMC7515561

[158] Krausz M Westenberg JN Vigo D Spence RT Ramsey D. Emergency response to COVID-19 in Canada: platform development and implementation for ehealth in crisis management. JMIR Public Health Surveillance. (2020) 6:e18995. 10.2196/18995 32401218 PMC7236607

